# Viral Hepatitis in Pakistan: Past, Present, and Future

**DOI:** 10.5005/jp-journals-10018-1172

**Published:** 2016-07-09

**Authors:** Amna Subhan Butt, Fatima Sharif

**Affiliations:** 1 Department of Medicine, Aga Khan University Hospital, Karachi, Pakistan

**Keywords:** Hepatitis A, Hepatitis B and C, Hepatitis E, Pakistan, Viral hepatitis.

## Abstract

Viral hepatitis is a major cause of morbidity and mortality worldwide and a rising cause for concern in Asian countries. Weather it is blood borne or water/food borne hepatotropic virus, increasing burden is alarming for Asian countries. In this review we have evaluated the existing data to estimate the burden of viral hepatitis in populations of all age groups nationwide, along with an assessment of the risk factors and preventive and management strategies currently employed in Pakistan. The aim of our work is to consolidate and supplement the present knowledge regarding viral hepatitis in light of past and present trends and to provide future direction to the existing health policies.

**How to cite this article:**

Butt AS, Sharif F. Viral Hepatitis in Pakistan: Past, Present, and Future. Euroasian J Hepato-Gastroenterol 2016;6(1):70-81.

## FOOD BORNE PATHOGENS: HEPATITIS A AND E

Developing countries are at a high risk of infection with orofecal pathogens. Likewise, in Pakistan poor sanitary conditions and lack of hygienic practices lead to 90% of children being infected with hepatitis A before reaching 10 years of age.^1-4^ Hepatitis A virus accounts for 50 to 60% of cases of acute viral hepatitis in the pediatric population of Pakistan.^5^ Moreover, up to 100% of children tested positive for HAV IgG by 14 years of age, indicating that most people are exposed to the disease during childhood (Table 1).^6^ Again as compared to children, 5.4 to 6.1% of adults have been found to have acute hepatitis due to HAV.^7^

**Table 1:** **Table 1:** Prevalence of hepatitis A and E in Pakistan

	*Study period*	*Sample size*	*Study site*	*Age mean ± SD (range)*	*Male n (%)*	*Female n (%)*	*Subjects*	*HAV IgM + n (%)*	*HAV IgG + n (%)*	*HEV IgM + n (%)*	*HEV IgG + n (%)*	*A + E n (%)*
Malik IA^52^	1985–1986	91	Rawalpindi	1–15	61 (67)	30 (33)	Children hospitalized with hepatitis	54 (59.3)	—	—	—	—
Haider Z^7^	1991–1991	93	3 tertiary care hospitals in Lahore	Adults = 32 Children = 7	70 (75.3)	23 (24.7)	Hospitalized patients with acute viral hepatitis	5 (5.4)	—	—	—	—
Agboatwalla M^53^	1990–1991	236	Karachi	—	—	—	Healthy Pakistani children	5.3	55.8	—	–	—
Qureshi H^11^	—	98	Various schools, well baby clinics, Karachi	7 months–10 years	—	—	Healthy children	—	80 (81.6)	—	18 (18.4)	17 (17.3)
Hamid SS^10^	—	233	Aga Khan University, Karachi	48.6 ± 11.4	142 (60.9)	91 (39.1)	Patients with CLD	—	228 (97.8)	—	41 (17.5)	—
Bryan JP^54^	1988	109	Abbottabad	28	109 (100)	0 (0)	Patients with acute jaundice	—	—	104 (95)	103/105 (98%)	—
Waheed-uz-Zaman T^23^	2003–2004	626	Armed Forces Institute of Pathology (AFIP), Rawalpindi	HAV IgM+ 3–27 years	—	—	Patients with clinical suspicion of hepatitis A	252 (40.57)	—	—	—	—
Aziz S^6^	2002–2004	380	Three selected squatter settlements of Karachi	5 months–15 years	—	—	Healthy children	—	100% in age of 14 years		26% in age of 14 years	
Bashir K^14^	2007–2007	93	Mayo Hospital, Lahore	30.95 ± 15.35	48 (52)	45 (48)	Patients with nonreactive serology for hepatitis B, C	—	—	5 (5.4)	4 (4.3)	—
Khan A^20^	2007–2008	89	Aga Khan University, Karachi	30.9 ± 14.5	43 (48.3)	46 (51.7)	Symptomatic patients with liver disease	4 (6.1)	—	53 (81.5)	—	—
Jafri W^15^	2008–2009	540	Karachi	1–15 years	—	—	Children from an urban slum locality, Karachi	—	—	13 (2.4)	78 (14.4)	—

What was previously thought to be an acute infection producing subclinical events in children has now proved to be a debilitating condition; an alarming number of hepatitis A related liver failure cases were reported in a recent study of 2,735 confirmed HAV cases out of which 36.7% died.^8^ The classical presentation of hepatitis A is in the form of vague abdominal symptoms, fever, and malaise, not necessarily accompanied by jaundice or hepatomegaly.^9^ It may remain silent in children and adults too. Hamid et al, studied 233 cases of chronic liver disease and 97.8% of those were found to be exposed to HAV.^10^ Due to variability in the presenting complaints, particularly the absence of jaundice, it is likely that the reported exposure rates of acute hepatitis A are much lower than the actual rate existing in the population.^11^

Hepatitis E in Pakistan has been witnessed to occur as outbreaks and sporadic cases in circumstances involving clustering of large numbers of people in areas where the water supply was contaminated.^12,13^ Overcrowding reflects poor sanitation and lifestyle in this patient population.^14^ A total of 14 to 26% of the apparently healthy pediatric population was found to be exposed to hepatitis E (HEV IgG reactive).^6,15^ Variability was found in reported prevalence of HEV in various studies. However, up to 20 to 22% of adults and 2.4% of children were found to have acute hepatitis due to HEV (Table 1). This disease has produced catastrophic effects in pregnant women, resulting in maternal mortality rates ranging between 20 and 29.3% and perinatal mortality rate of up to 30.3 per 1,000 live births.^16,17^ It is also a significant cause of mortality in patients with preexisting chronic liver disease.^18^ A nosocomial outbreak of hepatitis E in a neurosurgery ward in Karachi, which was attributed to incorrect sharing of intravenous administration sets between patients, has led to speculation that this pathogen may also be parentally transmitted, an idea that is supported by a few similar reports in the literature.^19^ This possibility needs to be looked into, in order to strengthen the current preventive strategies in place.

In light of the significant morbidity and mortality caused by HEV infection, it is imperative to have accurate means of detection, which would facilitate early diagnosis and management for better outcomes. However, limited sensitivity of the serological assay available in Pakistan hinders the prompt diagnosis and treatment of hepatitis E infections, especially at the time of outbreaks.^20^ At present HEV of two distinct origins has been identified in Pakistan based on genotype, namely Sar-55 from Central Asian origin and Abb-2B from South Asia. Of these, Abb-2B is believed to be endemic in Pakistan.^21^

## CHANGING TRENDS AND FUTURE DIRECTIONS

Few community-based studies have been conducted to estimate the incidence and prevalence of hepatitis A and E in Pakistan. The majority of reported data was from hospital settings and with small sample size. In general, HAV and HEV infection was found endemic in Pakistan and some changes in the disease pattern have been noticed over the last few years.^22^ Recent studies show that there is a changing trend in the occurrence of hepatitis A as now people of all age groups appear to be affected. The earlier studies revealed that by the age of 14 years almost 100% of children have been exposed to hepatitis A (reference 6, 23). However, during last few years increasing number of cases with acute hepatitis among adults has been observed^23^ A major source of infection has been recently identified with the isolation of HAV in fresh vegetables due to irrigation of fields with polluted water.^24^

In 2009, the Pakistan Field Epidemiology and Laboratory Training Program (FELTP) established a hepatitis sentinel surveillance system in collaboration with CDC’s Division of Viral Hepatitis and Ministry of Health, involving five public sector tertiary care hospitals, located in four provincial headquarters (Lahore, Peshawar, Karachi, and Quetta) and in Islamabad (the federal capital^25^). A total of 712 cases of viral hepatitis were reported from June 2010 to March 2011; 19.8% had acute hepatitis A and 12.2% had acute hepatitis E. Both HAV and HEV were more common in males, 69.5 and 72.4% respectively. A change in age distribution was noticed for hepatitis A; the highest prevalence of HAV was found in the age group 20 to 29 years (41.2%) followed by 30 to 39 years (16.3%) and 6 to 19 years (12.8%). While HEV was found in 30.4, 22.9, and 18.8% cases in the age group 20 to 29, 30 to 39, and 40 to 49 years, jaundice was found on presentation in 43.7% of HEV patients as compared to 28.4% of HAV patients. Drinking unboiled water was found to be the most important risk factor here.^25^ So, there is a changing trend of developing hepatitis A in the age beyond 18 years, which was not there in our patients previously due to universal immunity found against HAV by the age of 18. This might be some improvement in hygienic conditions or better understanding of parents to maintain good hygiene for children and the exposure to contaminated water and food in later years of life. However, this data carries certain limitations and could not be generalized. For instance, the catchment population was mainly urban poor visiting public hospitals in major cities; hence the study did not include private sector hospitals, smaller cities, rural areas, and patients with milder disease. Hence, there is an immense need to expand the program to find out better estimates. Beside immense need to improve sanitary conditions, availability of safe water, and educating public about modes of spread and preventive measures, the utilization of HAV vaccination will be helpful in reducing the disease burden.^9^ Early vaccination of children for HAV could reduce the associated morbidity and mortality. It is expected that vaccination against HEV at an early age will also help prevent acute infection. However, in data currently available we have not been able to find any study assessing the effectiveness of HAV vaccine in Pakistani population; this gap in our knowledge at present needs work in the future.

## BLOOD BORNE PATHOGENS: HEPATITIS B, C, AND D

According to statistics compiled by the World Health Organization (WHO), 2 to 5% of the Indian subcontinent is affected by hepatitis B whereas 4 to 5% of the Pakistani population is suffering from hepatitis C, resulting in one of the highest infection rates in the world.^26,27^ Almost one-third HBV-infected population in Pakistan were shown to be co-infected with hepatitis D virus, a defective RNA virus that requires the presence of HBV for replication.^28^

Occurrence of these blood borne infections has been assessed in the seemingly healthy population as well as in high-risk groups. A wide variability has been observed in the prevalence of hepatitis B and C in general the population and among people at risk. This is probably due to the difference in study settings, sampling frame and technique, and eligibility criteria. In various studies, overall HBV and HCV prevalence in blood donors and seemingly healthy adult population was reported between 0.84 and 6.9% and 0.19 and 22.2% respectively (Table 2). Moreover, co-infection with hepatitis B with C or D was tested in very few studies. In children the prevalence of HBV, HCV, and HBV-HCV co-infection was found in 1.8, 1.6, and 0.11% cases respectively.^29^

**Table 2:** **Table 2:** Prevalence of hepatitis B, C, and D among blood donors and healthy volunteers

	*Study period*	*Sample size*	*Study site*	*Age mean ± SD (range)*	*Male n (%)*	*Female n (%)*	*Subjects*	*HBsAg reactive*	*Anti–HCV + n (%)*	*HBsAg + anti–HCV + n (%)*	*HBsAg + anti–HDV Ab + n (%)*	*Hepatitis B, C, D n (%)*
Khadim MI^55^	1982	140	Khyber, lady reading Hospital, Peshawar	Highest in 21–40 years	—	—	Blood donors, volunteer medical students	Donors = 11.3 Students = 11.7				
Kakepoto GN^56^	1989–1993	51,257	AKUH, blood donation camps of Karachi and Hyderabad	34.2 ± 4.1 (16–68)	44,184 (86.2)	7,073 (13.8)	Blood donor	1,173/51, 257 = 2.28	198/16, 705 = 1.18	—	—	—
Ahmad N^57^	2004– 2004	300	Faisalabad	32 ± 20	232 (77.3)	68 (28.7)	Blood donors, screening camp	—	48 (16)	—	—	—
Khan A^58^	2011–2011	356	CMH, Quetta	21 ± 4	356	—	Male Blood donors	–	79 (22.2)	—	—	—
Malik IA^59^	1984–1996	630	PULSE, Army Medical College, Rawalpindi	Adults	—	—	Volunteer students = 60 Volunteers new recruits = 365	5.3% (HBc 12.2) 10.7% (HBc 33.2%)				
Khokhar N^44^	1998–2004	47,538	Shifa International Hospital, Islamabad	HBV + = 40.5 HCV + = 44	44,685 (94)	2,853 (6)	Healthy adult individuals presented for medical evaluation as a pre–employment criteria	1,221 (2.56)	2,528 (5.31)	92 (0.19)	—	—
Jafri W^29^	2003–2004	3,533	Households in Karachi	1–15	1,826 (52)	1,707 (48)	Children from low to middle socioeconomic class	65 (1.8)	55 (1.6)	3 (0.11)	—	—
Khan S^60^	2005–2006	245 + 290 = 535	Northern Pakistan	11–25 (36%) 25–50 (43.6%)	291 (54.4)	245 (45.7)	Earthquake affected communities	—	8 (3.26) + 16 (5.5)	—	—	—
Hakim ST^33^	2006–2007	4,000	Jinnah and Karachi university	18–30	—	4,000	Healthy volunteer	181 (4.5)	208 (5.2)	1 (0.025)	—	—
Noorali S^61^	2000–2006	4,000	Karachi	Age range 18–30	0 (0)	4,000 (100)	Female student volunteers	180 (4.5)	—	—	—	—
Shaikh FH^62^	2006–2007	450	Larkana Highest (58.2) in 20–40 years age	Adults	353 (78.4)	97 (21.6)	Healthy volunteers	—	30 (6.6)	—	—	—
Hyder O^38^	2007–2009	58,680	Punjab (community based) Highest in 40–50 years (11.3)	26 (16–59)	—	—	Healthy males, pre–employment screening	—	4,034 (6.9)	—	—	—
Rauf A^63^	2010	590	Swat, northern Pakistan Highest in 36–45 years	Adults	290	300	Healthy volunteers	5 (0.84)	52 (8.81)	00	—	—
Alam MM^39^	2005–2006	1,300	NIH, Islamabad highest in 30–40 years, lowest in 10–20 years age	23.5 ± 3.7 (8–53)	64%	36%	Individuals tested for HBV	—	—	—	—	—
Sheikh NS^64^	2004	11,900	Balochistan	Median age = 34.5 years, SEM 1.79	6,874 (57.7) HbsAg + = 875 (12.7)	5,026 (42.2) HbsAg + = 291 (5.8)	Residents of rural areas	1,166 (9.8)	875 (12.7%) males and 291 (5.8%) females	—	—	—
Zafar A^65^	2001–2008	396,348	AKUH, clinical laboratory	29.8 ± 12.5	66,261 (30)	18,954 (11)	Individuals tested for hepatitis B	85,215 (21.5)	—	—	—	—
Siddiqui TS^66^	2010	15,793	Pakistan Rangers (Punjab) Central Hospital Lahore	—	15,793 (100)	0 (0)	Healthy adult males serving in Pakistan Rangers Punjab	396 (2.82)	511 (3.64)	—	—	—
Khan F^67^	2009	950	Malakand, KPK Highest 29.13% in 46–60 years of age		157 (78.5) males	43 (21.5)	IDPs, volunteers	52 (5.47) Other markers 21.05%*	—	—	—	—
Aziz S^68^	2007–2008	573	Nausheroferoz, thatta (community based)	24.74 ± 14.41	273 (47) 67% +	301 (52.5)% 33% +	Apparently healthy, asymptomatic adults and children above 1 year of age not previously screened or vaccinated for HBV and HCV	Villages: 31 (7.0) Peri–urban: 4 (3.1)	Villages: 111 (28.6) Peri–urban: 5 (3.9)	—	—	—
Memon MR^69^	2009–2010	913	Ghulam Muhammad Mahar Medical College, Sukkur	40	572 (62.5)	341 (37.34)	Pts undergoing elective surgery	33 (3.61)	117 (12.8)	—	—	—
Fayyaz M^32^	2014	3,549	Ayub teaching hospital, Abbottabad	Highest among 16–50 years	1,914 (53.9)	1,635 (46.1)	Patients visiting for dental care	48 (32.7)	97 (66)	—	—	—

*HBSAg and HBeag + 52 (5.47%), anti-HBs/anti-HBe + 235 (24.74%), gradual increase in incidence with increasing age

Realizing the burden of the disease, in 2007–2008 the first national survey was conducted to estimate the prevalence of hepatitis B and C in Pakistan.^30^ A total of 47,043 individuals were tested and the overall prevalence of hepatitis B was 2.4% and of hepatitis C was 4.8%. The highest prevalence of hepatitis B was found in the province of Baluchistan (4.3%) followed by the province of Sindh (2.5%). On the contrary, the highest prevalence of hepatitis C was found in the province of Punjab (6.7%) followed by Sindh (5.0%). In addition there were pockets of up to 22% HCV prevalence in our country. Males were found to be affected more than females (2.9%). Increasing age, exposure to therapeutic injections, history of surgeries, hospitalization, blood transfusion, shaving by barbers in community, and being married were the factors found to be associated with higher risk to acquire hepatitis B and C.^30,31^

Prevalence also varies depending on the geographic distribution and various settings. In a study, 32.7% of individuals who visited for dental care were found to have HBV and 66% had HCV.^32^ Hepatitis C was the most common attributing factor for viral hepatitis and chronic liver disease (Table 3). Hepatitis D was found in 23.6 to 35% cases of hepatitis B (Table 4). Hepatitis B genotype D was the most common one (83.89) followed by genotype B + D (15.56%).^33^ While in the case of hepatitis C, genotype 3a was the most common genotype (51.44) followed by 3a with 3b (20.04), 3b (15.87), and 1b (4.81).^34^ Approximately 4.4% spouses of patients with hepatitis C infection were also found to have HCV in a study reported from a tertiary care hospital,^35^ which raises the question for interfamilial transmission of HBV and HCV. Being at risk, health care workers were screened and 2.4 to 5.6% were found infected by HBV and HCV and co-infection was found in 3.2% cases.^36^ A very high proportion of HCV were affected by hepatitis B and C when evaluated in Jamshoro, Sindh.^37^ Women were screened for hepatitis B and C during pregnancy in various studies, and the prevalence of hepatitis C was found much higher than hepatitis B (2.5–13.3% *vs* 1.2–3%) (Table 5). Again both HBV and HCV prevalence was much higher in patients who received multiple blood transfusions, who were on hemodialysis and IDUs (Table 6).

**Table 3:** **Table 3:** Prevalence of hepatitis B, C and D among patients with viral hepatitis and CLD

Haider Z7	1991–1991	93	3 teaching hospitals in Lahore	Adults = 32 Children = 7	70 (75.37)	23 (24.7)	Hospitalized patients with acute viral hepatitis	59 (63.44)	6 (6.5)	2 (2.2)	2 (2.2)	—
Ahmad W^70^	1987–2007	5,193	JPMC and PMRC Karachi HCV highest in 30–50 years	HBV highest in 20–40 years	3,247 (62.5)	1,946 (37.5)	Pts having viral hepatitis	1,691 (32.6)	2,896 (55.5)	3 (1.3)	—	—
Ali A^71^	2010–2011	790	Different areas of Waziristan	Range 3–60	485 (61.4)	305 (38.6)	Suspected hepatitis patients in areas of military operations	324 (70.3)	—	—	—	—
Khan J^72^	2011	845	PIMS, Islamabad	Highest in 21–40 years (43%)	Anti-HCV+: 71.3% HBsAg: 23.8%	Anti-HCV+: 84.2% HBsAg: 12%	Patients hospitalized or seen in OPD suspected for viral hepatitis	45 (5.3)	199 (23.5)	11 (1.3)	—	—
Qureshi H^73^	1996–1999	400 410	PMRC, Karachi	42 ± 13	263	137	Patients with CLD Healthy controls	98 17	302 18			
Bukhtiari N^74^	1999–2000	97	CMH, Rawalpindi	51.6 (16–75)	52 (53.6)	45 (46.6)	Patients admitted with CLD	24.7% HBc 61.1%	64.9%	34 of HBV + pts	—	—

**Table 4:** **Table 4:** Prevalence of hepatitis B, C, and D co-infection

Zuberi BF75	2003–2005	246	Civil Hospital and Lyari General Hospital, Karachi	26.7 ± 11.9	138 (56.1)	108 (43.9)	HbsAg + pts checked for HDV	246 (100)	—	—	66 (26.8)	—
Shaikh MA^76^	2003–2008	774	Chandka Medical College Hospital, Larkana	Males = 36.5 ± 14.39, females = 34.03 ±13.16	478 (57)	336 (43)	Adults with HBV related liver disorders	774 (100)	—	—	183 (23.6)	—
Majid A^77^	2004–2008	25,944	District, teaching hospital, bannu, KPK	Highest prevalence in 46–55 years	13,953 (53.7)	11,991 (46.3)	Patients visited medical wards and clinics	502 (1.93)	850 (3.27)	00	–	—
Baig S^78^	2006–2007	129	PMRC and JPMC Karachi	31.5 ±12.39	108 (84)	21 (16)	Pts with HBV infection	129 (100)	4 (3.1)	4 (3.1)	45 (34.9)	4 (3.1)
Das K^79^	2007–2007	73	JPMC Karachi	Males = 23.88 ± 10.96 Females = 36.84 ± 15.69	48 (65.8)	25 (34.2)	Pts having HbsAg +	73 (100)	—	—	23 (31.5)	—
Zaidi G^80^	2009–2010	200	Different regions in Punjab	42.5 ± 8.9	121 (60.5)	79 (39.5)	HbsAg + pts in Punjab	96 (48)	—	—	24 (30)	—
Mumtaz K^81^	Last 5 years	480	AKUH, Karachi, Isra University Hospital, Hyderabad	33 ± 12.53	398 (82.9)	82 (17.1)	HbsAg + and HBV DNA PCR + pts	480 (100)	—	—	169 (35.2)	—
Baig S^82^	–	472	ZMUH, PMRC	Highest 16–50 years	375 (79.5)	97 (20.5)	Patients evaluated for HBV	472	—	—	—	

**Table 5:** **Table 5:** Prevalence of hepatitis B, C, and D among health care workers and spouses of index patients and pregnant women

Sarwar J36	2006–2007	125	DHQ Hospital Abbottabad	Median age 41 (25–58)	83 (66.4)	42 (33.6)	Health care workers	7 (5.6)	7 (5.6)	4 (3.2)	—	—
Sarwar J^83^	2006–2007	125	Dist headquarter hospital, Abbottabad	Median 41 years (25–58)	83 (66.4)	42 (33.6)	Health care workers	3 (2.4)	3 (2.4)	4 (3.2)	—	—
Gorar ZA^84^	2012	657	Jamshooro	Highest in 46–55 (45.6)	53 (64.4)	28 (34.5)	HCW 227 (34.6%) reactive for HBv and HCV…81 cases selected	34 (42)	47 (58)	—	—	—
Khokhar N^35^	2001–2004	227	Shifa International Hospital, Islamabad	HCV+ = 44.2 ± 8.31 HCV – =	HCV + = 6 (60) HCV – =125 (57.6)	HCV+ = 4 (40) HCV- = 92 (42.4)	Spouses of HCV patients	—	10 (4.4)	—	—	—
Gul N^85^	2006–2007	500	NWFP	Highest in 25–35 years		500	Pregnant women	—	43 (8.9)	—	—	—
Khattak ST^86^	2008	5,607	Saidu Teaching hospital, Swat	Highest among 20–40 years	–	5,607	Pregnant women	77 (1.3)	141 (2.52)	5 (0.09)	—	—
Bibi S^46^	2010	3,078	Liaquat university hospital Hyderabad	28.7±4.9	–	3,078	Pregnant women	—	4.7%			
Kumari K^87^	2012	300	Sir Syed College of Medical Sciences and Trust Hospital, Karachi	30	0 (0)	300 (100)	Pregnant women	6 (2)	40 (13.3)	—	—	—

**Table 6:** **Table 6:** Prevalence of hepatitis B, C, and D in high-risk population

Rauf MU88	2011	141	Karachi	21.33 ± 9.28	141 (100)	0 (0)	Garbage collectors	22 (18.8)	8.5 (10)	—	—	—
Maan MA^89^	2010–2012	39,780	District Headquarter Hospital Faisalabad	49.7 ± 2.7	HCV +ve = 5,876 (67.14) HCV –ve = 16,095 (51.87)	HCV +ve = 2,875 (32.85) HCV –ve = 14,934 (48.12)	Pts visiting Sexually transmitted infections (STI) clinic	—	8,751 (21.99)	—	—	—
Khan S^90^		348	Three tertiary care hosp. KPK	40.9±5 (54915-75)	244	140	Patients on hemodialysis	—	112 (29.2) 90 (80.4) were RNA+	—	—	—
Ali I^91^		167	KPK	Highest in >50 years (22.22)	18/14=	8/63=	Pt on hemodialysis, Thalassemia, major surgery, dental procedure, IUDs		Anti-HCV and PCR +: 26 (15.57) Thalassemia: 6 (15%) Dialysis: 7 (28%) Major surgery: 2 (8) Dental surgery: 5 (14.28) IDUs: 6 (14.28)			
Achakzai M^92^	2004	50	Quetta	30		0 (0)	Street recruited Injection drug users	3 (6)	30 (60)	—	—	—
ur Rehman L^93^	2010	200	KPK	—	200	—	IDUs	—	63 (31.5) PCR + 48 (24)			

Increasing seropositivity with anti-HCV has been seen with advancing age; it is postulated that this is because of a greater lifetime exposure to unsafe injections, which is an important risk factor for HCV transmission in Pakistan.^38^ HBV is mainly afflicting people of lower socioeconomic class in rural areas.^39^ It is questionable whether there is a gender predilection to acquiring blood borne hepatitis infections. In some studies no particular gender predilection has been seen, some show a higher prevalence in males, whereas others show a higher prevalence in females.^40-42^ In one study where a higher frequency of males with HCV was observed as opposed to females, it was considered a reflection of males seeking and receiving health care more often than females in our society.^43^ On the contrary, the greater social freedom that males have, especially in rural areas, could also lead to increased exposure to these pathogens.^41^

Asymptomatic HBV and HCV can produce deranged LFTs including AST, ALT, and AP in apparently healthy individuals.^33^ However, a study of a large group of healthy adult males who underwent medical evaluation as part of their pre-employment requirements revealed that HBV and HCV can be present even in young age and with a normal ALT.^44^ Serological testing of preoperative patients who presented to the doctor with a variety of indications, such as cataract surgery and plastic surgery again showed high prevalence of blood borne hepatitis infections. It is recommended that routine screening of all preoperative patients for hepatitis should be mandatory to curb disease transmission through asymptomatic patients.^40,42^

The frequency of hepatitis infection is more common in rural population than in urban population and more in multiparas than primigravidas.^45^ Pregnant women are at risk for contracting blood borne infections because they often develop severe anemia or postpartum hemorrhage, incurring the need for blood transfusion, which might not be safe as standard international guidelines are not followed by all blood banks in Pakistan.^46^ A history of previous surgeries, which include cesarian section, laparotomy, and D and C, has been implicated as a major risk factor in this patient population.^46,47^ Seropositive mothers are not only under threat of the chronic sequelae of hepatitis themselves but also pose a risk to their care givers and their offspring through vertical transmission of the disease.^48^ A recent study conducted on a large scale found that children born to mothers who were HBsAg positive had an eight times greater risk of acquiring HBV and this risk increased to 17 times if the mother simultaneously tested positive for HBeAg. The same study conducted a follow-up of children receiving HBV vaccination at 6, 10, and 14 weeks of birth as per the current EPI schedule. It concluded that this regime is not very effective and an additional dose given at birth should be added to the national immunization policy.^49^

## CHANGING TRENDS AND FUTURE DIRECTIONS

The only available surveillance data from our country is the one reported by FELTP (2009–2011).^25^ Out of 712 patients, newly reported HCV patients were 53.2%, and 10.8% were newly reported HBV patients and majority of them were males (> 62%). The highest proportion of HBV was found in the age group 20 to 29 years (39%) followed by 30 to 39 years (26.0%) while almost equal distribution of HCV infection was found in the age group 20 to 49 years (24–25.6%). Only 21% of these patients had jaundice on presentation and only 5.2% were vaccinated for hepatitis B at some point of time. History of therapeutic injections, dental/surgical procedures, body piercing, and visit to beauty salon were found significant factors associated with hepatitis B and C.

Having sad that, population-attributable estimates for risk factors associated with hepatitis B and C have been calculated on data sets of the first national survey in Pakistan.^50^ In case of hepatitis B the attributable risk for therapeutic injections was 3.5% (95% CI 2.9–3.9), for reuse of syringes 2.7% (95% CI 2.2–3.1), for practice of being shaved by a barber 2.1% (95% CI 1.7–2.6), and for ear/nose piercing 1.4% (95% CI 1.2–1.7). In case of hepatitis C, the attributable risk was 11.3% (95% CI 10.5–11.7), 6.2% (95% CI 6.1–6.9), 7.9% (95% CI 7.1–8.2), 5.9% (95% CI 5.2–6.1), and 5% (95% CI 4.3–5.6) for therapeutic injections in the past 1 year, reuse of syringes, shaved by a barber shop, practice of ear/nose piercing among females, and tattooing respectively. This simply means that the burden of the disease could be reduced significantly if these factors could be controlled. Over the last few decades a switch in the distribution of hepatitis B and C has been observed. Hepatitis B was more prevalent and a leading cause of HCC in Pakistan in the 1980s and 1990s. While HCV replaced HBV in subsequent years and emerged as the most common cause of liver disease and HCC.^22,51^ The steps taken at the government level with the help of WHO, CDC, and various NGOs are vaccination of children and adults for hepatitis B, provision of treatment for hepatitis B and C via the prime minister program for hepatitis control, and raising awareness in public awareness campaigns; the world hepatitis day was also conducted. Medications for HBV like interferon alpha, pegylated interferon, entecavir, tenofovir, and lamuvidine are in the national essential medicines list or are subsidized by the government. Drugs for treating hepatitis C that are on the national essential medicines list or are subsidized by the government include interferon alpha, pegylated interferon, and ribavirin. Sofosbuvir is now available in Pakistan but patients have to bear the cost.

## CONCLUSION

Despite measures taken to control viral hepatitis in Pakistan, there is lot more to do. The burden of hepatitis A and E will not reduce unless basic facilities including safe water supply and better sanitary conditions will be available across the country. There is a need to establish a central registry for outbreaks, mortality related to hepatitis and liver diseases, HCC, and surveillance system for viral hepatitis needs expansion. Implementation of universal vaccination of newborns and high-risk groups for HBV and better compliance will reduce the burden of HBV in future. Again awareness among health care providers to avoid unnecessary therapeutic injections, safe blood transfusion services, use of auto-disable syringes, and better utilization of available resources are key steps in the prevention and management of hepatitis B and C.
